# Value of 3D Preoperative Planning for Primary Total Hip Arthroplasty Based on Biplanar Weightbearing Radiographs

**DOI:** 10.1155/2019/1932191

**Published:** 2019-03-10

**Authors:** Y. Knafo, F. Houfani, B. Zaharia, F. Egrise, I. Clerc-Urmès, D. Mainard

**Affiliations:** ^1^Service de Chirurgie Orthopédique, Traumatologique et Arthroscopique, Hôpital Central, CHRU Nancy, Av de Lattre de Tassigny, 54000 Nancy, France; ^2^Pôle des Structures de Soutien à la Recherche, CHRU Nancy, Av de Lattre de Tassigny, 54000 Nancy, France

## Abstract

Two-dimensional (2D) planning on standard radiographs for total hip arthroplasty may not be sufficiently accurate to predict implant sizing or restore leg length and femoral offset, whereas 3D planning avoids magnification and projection errors. Furthermore, weightbearing measures are not available with computed tomography (CT) and leg length and offset are rarely checked postoperatively using any imaging modality. Navigation can usually achieve a surgical plan precisely, but the choice of that plan remains key, which is best guided by preoperative planning. The study objectives were therefore to (1) evaluate the accuracy of stem/cup size prediction using dedicated 3D planning software based on biplanar radiographic imaging under weightbearing and (2) compare the preplanned leg length and femoral offset with the postoperative result. This single-centre, single-surgeon prospective study consisted of a cohort of 33 patients operated on over 24 months. The routine clinical workflow consisted of preoperative biplanar weightbearing imaging, 3D surgical planning, navigated surgery to execute the plan, and postoperative biplanar imaging to verify the radiological outcomes in 3D weightbearing. 3D planning was performed with the dedicated hipEOS® planning software to determine stem and cup size and position, plus 3D anatomical and functional parameters, in particular variations in leg length and femoral offset. Component size planning accuracy was 94% (31/33) within one size for the femoral stem and 100% (33/33) within one size for the acetabular cup. There were no significant differences between planned versus implanted femoral stem size or planned versus measured changes in leg length or offset. Cup size did differ significantly, tending towards implanting one size larger when there was a difference. Biplanar radiographs plus hipEOS planning software showed good reliability for predicting implant size, leg length, and femoral offset and postoperatively provided a check on the navigated surgery. Compared to previous studies, the predictive results were better than 2D planning on conventional radiography and equal to 3D planning on CT images, with lower radiation dose, and in the weightbearing position.

## 1. Introduction

The goals of total hip arthroplasty (THA) for the treatment of osteoarthritis are the reduction of pain and restoration of normal function, permitting a return to the patient's normal activities. To reduce pain and restore normal function after total hip arthroplasty (THA), it is imperative that the implant positioning respects recognized quality criteria, including maintenance of leg length and femoral offset, good implant orientation (anteversion and inclination of the cup, anteversion of the femoral stem), and a suitable size of the implants. When these are not respected, complications can lead to residual pain, instability, premature prosthetic wear, and difficulties in walking, causing patient dissatisfaction [1–9]. More accurate cup and stem sizing has the potential to reduce surgical time and inventory needs and provides a double-check on the size chosen intraoperatively.

While navigation has been shown to achieve a surgical plan precisely, the choice of that plan remains key to surgical success. Navigation performed on a supine patient ignores weightbearing influences and typically depends only on intraoperative data rather than preoperative planning so that sizing or surgical difficulties cannot be predicted in advance. Furthermore, leg length and offset are rarely checked postoperatively.

**Figure 1:**
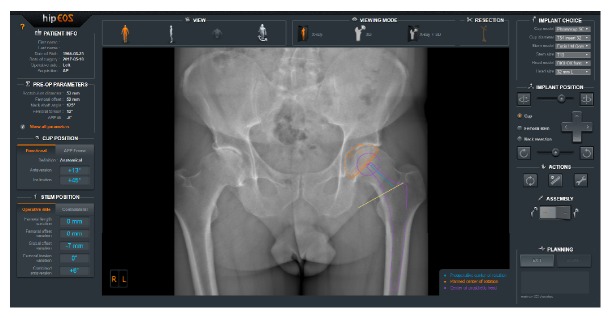
Screenshot from hipEOS® planning software.

**Figure 2:**
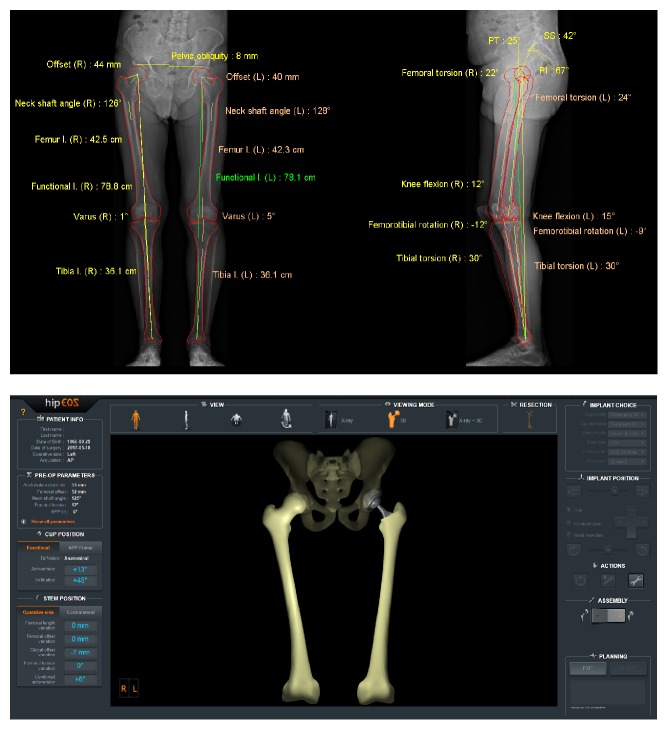
EOS images after modeling, including clinical parameters generated automatically (top) and 3D model of the surgical plan (bottom).

The hipEOS® planning software (EOS imaging, Paris, France) (Figure 1) is based on radiographs obtained by the EOS® imaging system (EOS imaging, Paris, France) (Figure 2). This imaging system rests on the simultaneous acquisition, in the standing, weightbearing position, of two orthogonal radiographic images using slot-scanning technology. The EOS imaging system provides a novel biomechanical and morphological approach in a functional position with the advantage of very low radiation dose [10–12] in contrast to computed tomography (CT), which is acquired in a supine position with a high radiation dose. Scanning of the entire body at one time also avoids the effects of stitching, magnification, and scale distortion of a long leg radiograph. The accuracy of the EOS imaging system has been demonstrated in the literature compared to conventional radiographs and CT [13, 14], making it an excellent tool for quality control of the surgery. 

The preoperative images obtained are subsequently modeled in 3D using sterEOS® software. The hipEOS software then integrates the manufacturers' 3D component templates into the modeled bones. To our knowledge, a prospective study does not exist studying the value of this new planning technique.

The principal objective of this pilot study was therefore to investigate the capability of the EOS solution (EOS images plus hipEOS planning) to predict and control THA parameters by comparing: the planned component size with that implanted and the difference between the planned leg length and offset and that measured postoperatively on EOS images.

## 2. Materials and Methods

In this prospective, monocentric, pilot study, we included all patients operated on consecutively at our institution over 24 months, meeting our inclusion criteria, between November 2014 and November 2016, by a senior surgeon (DM). The study respected the ethical standards for biomedical research in agreement with the Declaration of Helsinki (latest revision, 2013) and met the ethical review requirements for our institution.

All of the patients presenting with primary hip osteoarthritis were included. Excluded were patients presenting with hip osteoarthritis with a dysplastic acetabulum or femur, patients for whom a dual-mobility cup was planned, those with previous surgery of the acetabulum, patients without preoperative or postoperative EOS images, those with a previous total hip or knee arthroplasty, and those with a revision THA.

Each of the patients received biplanar preoperative EOS images 1 month before surgery and postoperatively between 4 to 8 months after surgery. The exams were all acquired as part of the standard routine for THAs in our department or were acquired for this study. A radiologist reconstructed the three-dimensional (3D) models of the femur, tibia, and pelvic parameters using the sterEOS software.

Planning was performed preoperatively by the surgeon using the hipEOS software (version 2.6), based on the 3D reconstructed models; the anonymized results were saved automatically on the provider's secure servers. Using the hipEOS software, the size of the femoral stem, size of the acetabular cup, the change in leg length, and change in femoral offset were determined for the surgical plan.

Patients were all operated on by an antero-lateral Hardinge, mini-invasive surgical approach, in the lateral decubitus position at 45°, using the Orthopilot™ navigation system (BBraun, Melsungen, Germany). The prostheses used were an uncemented femoral stem (Excia™, BBraun, Melsungen, Germany) and an uncemented, impacted cup (Plasmacup™ SC, BBraun, Melsungen, Germany), resulting in a ceramic-ceramic wear couple.

The implant positioning objectives were to maintain leg length within ±5 mm, femoral offset within ±5 mm, and, for the cup, positioning within the Lewinnek safe zone: 40° ±10° of inclination and 15°±10° of anteversion, relative to the patient's functional weightbearing plane.

Statistical analysis was performed by our institution's Clinical Research Support group. Analyses were conducted using the SAS software, version 9.4 (SAS Institute, Inc, Cary, N.C.). The results are presented as absolutes for the data collected, as percentages for the discrete variables (acetabular cup and femoral stem sizes), and as means, standard deviations, median, quartiles, and extremes for the continuous variables (femoral offset and leg length). Paired Student t-tests were used to evaluate differences. The threshold for statistical significance was fixed at 0.05. To verify the agreement between the planning and the postop images, a Kappa coefficient was calculated. Accuracy was defined as the difference between the planned value and the value measured on the postoperative EOS images, i.e., the preop-postop agreement.

## 3. Results

120 patients were operated on during the inclusion period. Of these, 87 were excluded: 20 patients having a revision, 20 patients already having a total hip or total knee prosthesis, 15 presenting with acetabular dysplasia, 2 with previous acetabular surgeries, 20 patients who did not have a preoperative or postoperative EOS image, and 9 patients with a dual-mobility cup. One patient was lost to follow-up.

The mean age of the 33 included patients was 65 years (SD: 14, range 32-84 years), BMI 27 kg/m^2^ (SD: 4, range 22-36), with more women than men (19W, 14 M); 23 were operated on the right side, 10 on the left.

The collected data are summarized in Table 1. Mean femoral stem size was 12 (SD: 2, range 8-15) and mean acetabular cup size was 52 (SD: 3, range 46-56).

**Figure 3:**
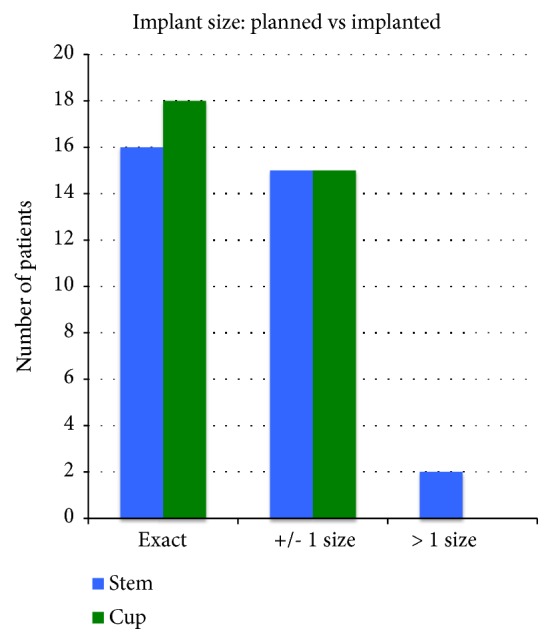
Comparison of planned versus implanted femoral stem and acetabular cup sizes. 100% of acetabular cups and 94% of femoral stems were within 1 size.

The planned femoral stem size corresponded with that implanted ± one size in 31 of 33 cases (94%) (Figure 3). The planned acetabular cup size corresponded with that implanted ± one size in 33 of 33 cases (100%) (Figure 3).

The planned femoral stem size corresponded exactly with that implanted in 16 of 33 cases (48%) (Figure 3) and in 18 of 33 cases (55%) for the acetabular cup (Figure 3).

The planned size of both the femoral and acetabular components corresponded with that implanted ± one size in 31 of 33 cases and exactly in 10 out of 33 cases.

The planned stem size averaged 11.4 (SD: 1.4) versus the implanted stem size averaging 11.7 (SD: 1.7). There was no significant difference between the planned versus implanted size (p=0.06). The planned cup size averaged 51.0 (SD: 3.0) versus the implanted cup size averaging 51.6 (SD: 3.0). These were significantly different (p=0.02).

The mean leg length difference planned was 1.9 mm (SD: 2.0) and the mean leg length difference measured by EOS postoperatively was 3.8 mm (SD: 5.7). There was no significant difference (p=0.07). The difference between the planned value and that measured on the postop EOS images was therefore -1.9 mm (SD: 5.9).

The mean offset difference planned was 0.6 mm (SD: 4.2) and that measured postoperatively by EOS was 0.3 mm (SD: 5.0). There was no significant difference (p=0.78). The difference between the planned value and that measured on the postop EOS images was therefore 0.3 mm (SD: 5.6).

No complications occurred during this series.

## 4. Discussion

This prospective pilot study evaluated the value of preoperative planning of primary total hip arthroplasty by the EOS system, using the hipEOS software.

The study was conducted at a single centre and the patients were operated on by a single surgeon. This provided homogeneity in the surgical technique and in the type of implant used. In addition, to ensure a homogeneous series, we excluded patients suffering from osteoarthritis with a dysplastic acetabulum or femur, as well as those with previous acetabular surgery and revision surgeries. Patients with a previous total hip or knee were also excluded because the 3D modeling performed with the sterEOS program cannot currently be used in the presence of implants, due to the occlusion of key landmarks. Some patients did not have a preoperative EOS scan due to unavailability of the machine or due to the patient declining to acquire the scan.

The current use of 2D planning consists of numerous approximations because it cannot reliably take into account knee flexion or femoral rotation, which induces measurement errors in the leg length [15], the planned implant size, and femoral offset [16, 17].

The EOS imaging system consists of a simultaneous acquisition in the weightbearing standing position of two orthogonal radiographic images of the entire lower limb and pelvis thanks to the linear slot-scanning, with a very low radiation dose [10–12], especially in comparison with CT [12]. 3D modeling by the sterEOS software therefore allows accuracy approaching that of CT [13, 14]. Linear slot-scanning of the entire body is achieved in 20 seconds.

For the last 4 years, all of the patients operated on for a total hip arthroplasty at our institution benefit from a preoperative and postoperative EOS scan. It allows a preoperative evaluation of the anatomy of the patient: leg length, pelvimetry, native femoral neck orientation, and femoral offset. Postoperatively, the images allow a quality control for the position and orientation of the implants, the leg length, and the femoral offset. EOS images have a very good accuracy [13, 14, 18], and the ultimate goal is to no longer use the more radiating conventional radiography.

The follow-up time to perform the postoperative imaging was set at least 4 months after the surgery so that the patient could reestablish a stable state.

The use of navigation allows intraoperative control of the positioning of the implants [19] and constitutes an additional aid for the surgeon to achieve the desired objectives.

The selection of an appropriately sized implant is essential. Femoral stem size was determined by the size of the femur and by the ideal position chosen during the planning. This position depends on the type of metaphyseal or diaphyseal contact and the shape of the implant, the type of fixation with or without cement, and the presence or not of a collar. If an implant without cement is chosen, as in this study, the stem size must be sufficiently large to allow primary stability following “press-fit” impaction. A femoral stem that is too large could lead to intraoperative complications such as a femoral fracture. This could likewise lead to leg lengthening by suspension of the stem. By contrast, a stem that is too small could subside and lead to prosthetic instability through a cam effect. In the case of cemented implants, premature loosening of the femoral stem is possible by increasing the stresses and by micromovements of the implant and the cement. In a clinical study with followup of at least 20 years [20], undersized stems, defined as filling the medullary canal by less than 80%, multiply the risk of aseptic loosening by 4.2.

The size of the acetabular implant depends on the size of the acetabulum while also allowing ideal positioning. This position should not move the centre of rotation of the hip in the three planes. It likewise depends on the characteristics of the cup: thickness, associated screws, and fixation method with or without cement. If a noncemented cup is used, as in this study, the size must permit primary stability following “press-fit” impaction. An undersized cup can lead to instability of the cup. By contrast, a cup that is too large can lead to intraoperative fractures of the acetabulum. Odri et al. [21] found a significant increase in the risk of postoperative pain in the case of overdimensioning.

The size planning of the components gave excellent results with 94% accuracy within one size for the femoral stem and 100% accuracy within one size for the acetabular cup.

The proximal femur of one of the patients was considerably larger than the canal, so a larger size was chosen to fill the proximal femur; the postop X-ray did not show suspension of the stem.

Several studies have shown that the use of cemented femoral stems provides more reliable planning of implant size because a primary stability can be easily obtained with this type of fixation [22].

Sariali et al. [23] found comparable results for CT planning. The study provided evidence regarding the lack of reliability of 2D planning with 43% accuracy for the cup and femoral stem.

The results of the present study are even better than those of a previous retrospective pilot study by our group, which showed significantly better results for 3D planning of the stem size based on biplanar low dose radiographs versus conventional 2D planning (84% for 3D versus 68% for 2D; p=0.04) [24]. The further improvements in the accuracy of the present results are probably due to the experience of the surgeon after the learning curve and to a newer version of the software. Subsequent software improvements (now at version 3.0) and range-of-motion analysis could improve the accuracy even further.

The consequences of leg length discrepancy (LLD) are first and foremost clinical, especially when it is perceived by the patient, which can lead to dissatisfaction [9]. The limit of tolerance is set at 10 mm, because above this level symptoms are more frequent [1, 9]. Lengthening is clearly less well tolerated than shortening [1]. Below one centimeter, the consequences are variable from one patient to another [9].

The variations in length observed after surgery were in some cases rendered necessary in order to place an appropriately sized implant having a primary stability in relation to the level of the cut. Increasing the size, which is sometimes necessary to obtain a “press-fit”, can therefore lead to a resultant lengthening of the limb. This is likewise the reason for which we planned the change in the leg length with the objective of ±5 mm rather than exact.

The results of the study showed a difference between the planned leg length difference values and surgical result of -1.9±5.9 mm, which is comparable to the conclusions of Sariali et al. [25] with a mean planning accuracy of leg length of -1.8±3.6 mm (-8 to +4 mm) with CT and 1.4±6.4 mm (-9 to +13 mm) in 2D.

A reduction in offset has multiple consequences [5, 6, 16]: gait instability, limping, which could require a walking aid due to limited function of the gluteus medius muscle, limited articular range of motion, dislocation due to a cam effect [6], and accelerated wear of the polyethylene [7]. The tolerance limit is classically set at a 15% reduction. Increased offset is less well known, some studies not finding an influence on hip mobility [8].

The agreement between the postop result and the hipEOS plan for femoral offset averaged 0.3 mm (SD ± 5.6), which appears to be slightly better than the results of Sariali et al. [25] who obtained a mean planning accuracy of femoral offset of 1.3±2.6 mm (-4 to +6 mm) by CT and of -0.9±5.7 mm (-13 to +9 mm) in 2D. These results demonstrate that the use of the EOS system from preop to postop, i.e., planning, execution of the planning in the operating room, and postoperative control, allows the surgeon to predict and to perform quality control on implant size, leg length, and femoral offset which are important parameters of THA success.

This study has some limitations. The number of subjects was relatively small. As a pilot study, it was insufficiently powered to prove the lack of statistical differences; however, the number was sufficient to demonstrate the reliability of the software. Furthermore, in contrast to the previous retrospective study [24], there was no control group (e.g., 2D planning or no planning) for comparison. The method also requires access to an EOS imaging system, which is not available at all centres. Other methods for determining leg length and offset discrepancies include mechanical devices and fluoroscopy, although they can be influenced by leg or hip rotation, which the 3D modeling helps to avoid. Differences in leg length and offset between planning and postop may be affected by the navigation system and surgical technique in addition to the preoperative planning.

## 5. Conclusion

This study demonstrated that the hipEOS software is a new tool in the surgeon's arsenal for 3D planning, with excellent reliability. Planning is made from EOS images, which have the advantage of being low dose and conducted under weightbearing, in the standing position, and can be performed postoperatively to check the results of the surgery. Compared to previous literature, including a pilot retrospective study by our group, the sizing results were better with 3D planning than 2D planning [24] and had comparable agreement to CT for changes in leg length and offset [25]. Preoperative planning and postoperative verification offer a valuable expansion on the capabilities of navigated surgery.

## Figures and Tables

**Table 1:** Global description of data collected.

	N	mean	SD*∗*	median	Q1	Q3	min	max
Size of femoral component implanted	33	11.7	1.7	12	11	13	8	15
Size of acetabular component implanted	33	51.6	3.0	52	50	54	46	56
Preop anatomical leg length (mm)	33	778.3	54.6	777	739	824	664	866
Postop anatomical leg length (mm)	33	782.2	55.2	780	741	827	663	874
Preop-postop change in leg length on EOS (mm)	33	3.8	5.7	3.0	-1.0	8.0	-6.0	14.0
Preop femoral offset (mm)	33	40.9	6.1	42.0	35.0	46.0	28.0	53.0
Postop femoral offset (mm)	33	41.2	5.2	40.0	37.0	45.0	34.0	52.0
Preop-postop change in offset on EOS (mm)	33	0.3	5.0	0.0	-4.0	4.0	-9.0	11.0
Size of femoral component planned	33	11.4	1.4	11	11	13	8	14
Size of acetabular component planned	33	51.0	3.0	50	48	54	46	58
Difference in leg length from planned length (mm)	33	1.9	2.0	2.0	0.0	3.0	-3.0	6.0
Difference in offset from planned offset (mm)	33	0.6	4,.	1.0	-1.0	3.0	-9.0	10.0

*∗* standard deviation.

## Data Availability

The data used to support the findings of this study are included within the article.
